# Comparison of an Inside Stent and a Fully Covered Self-Expandable Metallic Stent as Preoperative Biliary Drainage for Patients with Resectable Perihilar Cholangiocarcinoma

**DOI:** 10.1155/2022/3005210

**Published:** 2022-07-05

**Authors:** Hiroshi Mori, Hiroki Kawashima, Eizaburo Ohno, Takuya Ishikawa, Kentaro Yamao, Yasuyuki Mizutani, Tadashi Iida, Masanao Nakamura, Masatoshi Ishigami, Shunsuke Onoe, Takashi Mizuno, Tomoki Ebata, Mitsuhiro Fujishiro

**Affiliations:** ^1^Department of Gastroenterology and Hepatology, Nagoya University Graduate School of Medicine, Nagoya, Japan; ^2^Department of Endoscopy, Nagoya University Hospital, Nagoya, Japan; ^3^Division of Surgical Oncology, Department of Surgery, Nagoya University Graduate School of Medicine, Nagoya, Japan; ^4^Department of Gastroenterology, Graduate School of Medicine, University of Tokyo, Tokyo, Japan

## Abstract

**Background:**

There is a need for a more tolerable preoperative biliary drainage (PBD) method for perihilar cholangiocarcinoma (PHCC). In recent years, inside stents (ISs) have attracted attention as a less suffering PBD method. Few studies have compared IS with a fully covered self-expandable metallic stent (FCSEMS) as PBD for resectable PHCC. The aim of this study is to compare them.

**Methods:**

This study involved 86 consecutive patients (IS: 51; FCSEMS: 35). The recurrent biliary obstruction (RBO) rate until undergoing surgery or being diagnosed as unresectable, time to RBO, factors related to RBO, incidence of adverse events related to endoscopic retrograde cholangiography, and postoperative complications associated with each stent were evaluated retrospectively.

**Results:**

There was no significant difference between the two groups in the incidence of adverse events after stent insertion. After propensity score matching, the mean (SD) time to RBO was 37.9 (30.2) days in the IS group and 45.1 (35.1) days in the FCSEMS group, with no significant difference (*P*=0.912, log-rank test). A total of 7/51 patients in the IS group and 3/35 patients in the FCSEMS group developed RBO. The only risk factor for RBO was bile duct obstruction of the future excisional liver lobe(s) due to stenting (HR 29.8, *P*=0.008) in the FCSEMS group, but risk factors could not be indicated in the IS group. There was no significant difference in the incidence of bile leakage or liver failure. In contrast, pancreatic fistula was significantly more common in the FCSEMS group (13/23 patients) than in the IS group (3/28 patients) (*P* < 0.001), especially in patients who did not undergo pancreatectomy (*P*=0.001).

**Conclusions:**

As PBD, both IS and FCSEMS achieved low RBO rates. Compared with FCSEMS, IS shows no difference in RBO rate, is associated with fewer postoperative complications, and is considered an appropriate means of PBD for resectable PHCC. This trail is registered with UMIN000025631.

## 1. Introduction

Percutaneous transhepatic biliary drainage has been performed to provide preoperative drainage in patients with perihilar cholangiocarcinoma (PHCC) [1–3]. However, because there is a possibility of complications such as vascular injury, fluid, and electrolyte loss due to temporary extracorporeal drainage and metastasis seeding associated with percutaneous transhepatic biliary drainage [4–6], endoscopic biliary drainage is recommended initially for patients with resectable PHCC [7]. Conventional stents inserted across the sphincter of Oddi and endoscopic nasobiliary drainage were performed for endoscopic biliary drainage. It has been reported that drainage tube obstruction with cholangitis is significantly more common in conventional stents than in endoscopic nasobiliary drainage and percutaneous transhepatic biliary drainage [8, 9]. Furthermore, compared with conventional stents, endoscopic nasobiliary drainage can be used to monitor the amount and nature of bile [10] and is less likely to cause cholangitis [8, 11]. However, endoscopic nasobiliary drainage is inferior in that it causes more patient suffering and impaired enterohepatic circulation of bile [3, 12, 13]. Thus, it is necessary to explore methods of preoperative biliary drainage (PBD) other than conventional stent and endoscopic nasobiliary drainage.

The efficacy of the inside stent (IS), a plastic stent placed above the sphincter of Oddi, for patients with unresectable PHCC has been assessed [14]. Kobayashi et al. reported a mean stent patency of 85.2 days when plastic stents were placed in the bile duct of patients with resectable malignant hilar biliary obstruction [15], suggesting that it is as suitable as PBD for PHCC [16]. On the other hand, there are several reports on the efficacy of a fully covered self-expandable metallic stent (FCSEMS) as biliary drainage for unresectable malignant distal biliary obstruction [17, 18]. It was reported that FCSEMS is also effective in resectable PHCC [19], but none of the relevant reports include a large sample size, precluding a definitive conclusion.

In this single-center, retrospective study, short-term outcomes were compared between patients who received IS versus FCSEMS prior to surgery for PHCC.

## 2. Methods

The primary endpoint of this study was the rate of recurrent biliary obstruction (RBO) until undergoing surgery or being diagnosed as unresectable. The secondary endpoints were time to RBO, factors related to RBO, complications related to endoscopic retrograde cholangiography (ERC), and postoperative complications.

### 2.1. Study Design

Cases were collected at a single center and reviewed retrospectively. This study was approved by the Institutional Review Board of Nagoya University Hospital (No. 2016-0032) and was performed according to the guidelines set forth in the Helsinki Declaration for biomedical research involving human subjects (clinical trial registration number: UMIN000025631).

### 2.2. Patients

This study included patients diagnosed with suspected resectable PHCC based on multidetector-row computed tomography and treated with the IS or FCSEMS as final scheduled preoperative biliary drainage (FSPBD) between May 2017 and August 2019 at Nagoya University Hospital. In resected cases, the patient was diagnosed with PHCC on postoperative pathology; in unresected cases, the patient was diagnosed with PHCC after at least one year of clinical follow-up. The following exclusion criteria were applied: (i) difficulty in using the endoscopic approach to the duodenal papilla; (ii) Eastern Cooperative Oncology Group performance status 2–4; (iii) percutaneous transhepatic biliary drainage before FSPBD; (iv) FSPBD accompanied by endoscopic nasobiliary drainage; and (v) FSPBD using both IS and FCSEMS.

### 2.3. Procedures

The most appropriate surgical procedure was planned based on multidetector-row computed tomography, evaluated the patient's condition (presence of obstructive jaundice or cholangitis), and then performed ERC. The endoscope used was a JF260V™ or TJF260V™ (Olympus Medical Systems Corporation, Tokyo, Japan); VisiGlide2™ (Olympus Medical Systems Corporation, Tokyo, Japan), EndoSelector™ (Boston Scientific Japan, Tokyo, Japan), or M Through™ (Medicos Hirata, Osaka, Japan) was used as the guidewire. During ERC, malignancy and longitudinal tumor progression were assessed by intraductal ultrasonography [20] and biliary forceps biopsy [21, 22], and drainage of the future remnant liver lobe(s) was performed. Endoscopic nasobiliary drainage was selected when the total bilirubin level was more than 3 mg/dl [23], and IS or FCSEMS was used instead when the total bilirubin level was less than 3 mg/dl if the waiting period before surgery was expected to exceed two weeks.

Stents were placed in the left bile duct in cases of planned right hepatectomy, in the right bile duct in cases of left hepatectomy, in the left lateral sectional bile duct in cases of right hepatic trisectionectomy, and in the right posterior sectional bile duct in cases of left hepatic trisectionectomy. When a stent had been placed in the excisional liver lobe at a previous hospital, we placed stents not only in the future remnant liver as described above but also in the previously inserted bile duct.

IS and FCSEMS are indicated for patients in whom cholangiography shows that the lower end of the stenosis is more than 20 mm from the papilla. When the confluence of the first bile duct on the future remnant liver lobe(s) is more than 5 mm from the upper end of the stenosis, FCSEMS is indicated, and when it is less than 5 mm, IS is indicated.

**Figure 1:**
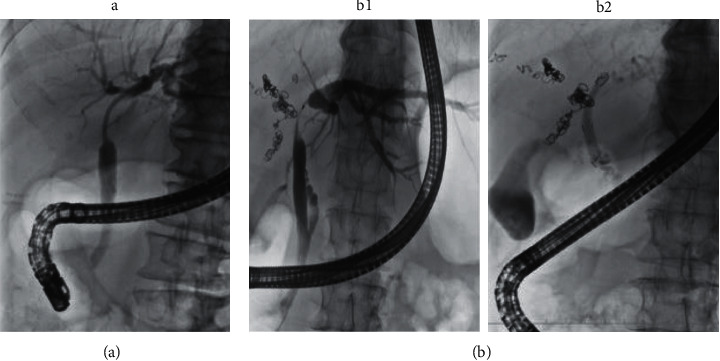
Placement of each stent. (a) The IS case. A patient with Bismuth-Corlette type IIIa PHCC for whom right hepatectomy was the expected surgical operation. Because B2, B3, and B4 confluence almost simultaneously, the IS (7 Fr 9 cm deep angle) was placed in the left bile duct. (b) The FCSEMS case. A patient with Bismuth-Corlette type IIIa PHCC for whom right hepatectomy was the expected surgical operation. The cholangiogram shows that the confluence of B4 is more than 5 mm from the upper end of the stenosis (b1); FCSEMS (6 mm × 4 cm) was placed such that B4 was not obstructed (b2). IS, inside stent; FCSEMS, fully covered self-expandable metallic stent; PHCC, perihilar cholangiocarcinoma; B2, left lateral superior segmental bile duct; B3, left lateral inferior segmental bile duct; B4, left medial segmental bile duct.

In this study, FCSEMS (Niti-S™, 6 mm × 40 mm, Century Medical Inc., Tokyo, Japan, or Hanaro™, 6 mm × 60 mm, Boston Scientific Japan, Tokyo, Japan) was used depending on the stenosis length. The FCSEMS was placed above the papilla in such a way that the upper end of the stent would not obstruct the bile duct branch of the future remnant liver lobe(s), and the lower end of the stent would be more than 20 mm above the papilla. The IS was placed with its upper end in the bile duct of the future remnant liver lobe(s) and the lower end more than 20 mm above the papilla. An IS with a preloaded thread, Amsterdam type (Through and Pass™ 7 Fr 9 cm deep or light angle, Gadelius Medical K. K, Tokyo, Japan), was used. Deep-angle stents were used when the bending from the hilar bile duct to the inserted bile duct was strong and light-angle stents when the bending was weak (Figure 1).

When RBO occurred after stenting, endoscopic treatment was performed immediately. The IS or FCSEMS was grasped with forceps and removed, and endoscopic nasobiliary drainage was placed in the same bile duct branch and/or in the undrained bile duct branch of the future excisional liver lobe (s).

### 2.4. Definitions

Adverse events after ERC, such as cholangitis, cholecystitis, and pancreatitis, were assessed based on Tokyo criteria 2014 [24].

RBO is defined as the redilatation of intrahepatic bile ducts with elevated hepatobiliary enzyme levels and includes segmental cholangitis in undrained areas (bile duct branch of future excisional liver lobe). Patients who did not develop RBO until surgery or those who were diagnosed as unresectable were considered censored cases. Time to RBO was defined as the time between FSPBD and RBO.

Factors affecting RBO in both groups were evaluated. Stenosis length was defined as the distance from the lower end to the upper end of the stenosis in the bile duct branch of the future remnant liver lobe(s). Obstruction of bile duct branches by stenting was defined as stent occlusion of the bile duct branch of the future excisional liver lobe(s) that could be opacified at ERC. The distance from the stenosis to the branch was defined as the distance between the upper end of the stenosis and the confluence of the first branch of the future remnant liver lobe(s).

Bile leakage, liver failure, and pancreatic fistula were examined according to the International Study Group of Liver Surgery and International Study Group of Pancreatic Surgery. Grades B and C, which require active therapeutic intervention, were assessed as postoperative complications [25–27]. Bile leakage and liver failure were classified as with or without hepatectomy and pancreatic fistula as with or without pancreatectomy.

### 2.5. Statistical Analysis

Statistical calculations were performed using SPSS 27.0 (SPSS, Chicago, Illinois, USA). The analyses were performed using the Mann–Whitney *U* test for continuous variables and the chi-squared test for categorical variables. Continuous parameters are presented as medians (interquartile range). For those with different backgrounds, the evaluation was performed using propensity score matching. Time to RBO was calculated using the Kaplan–Meier method and the log-rank test.

For patients in each group who required reintervention due to RBO after FSPBD, risk factors for RBO were calculated by univariate analysis and then examined by Cox proportional hazard analysis; multivariate analyses included factors with *P* < 0.2 in univariate analysis. The analysis also checked for noncollinearity among these factors.

## 3. Results

### 3.1. Patient Data

This study included 86 patients (IS group, 51; FCSEMS group; 35). Bismuth-Corlette classification included I/II and III/IV, with the latter being more common in the IS group than in the FCSEMS group (*P* < 0.001). The final diagnosis was bile duct carcinoma in 72 patients and gall bladder carcinoma in 14 patients, with no significant difference between the two groups (*P*=0.439) (Table 1).

**Figure 2:**
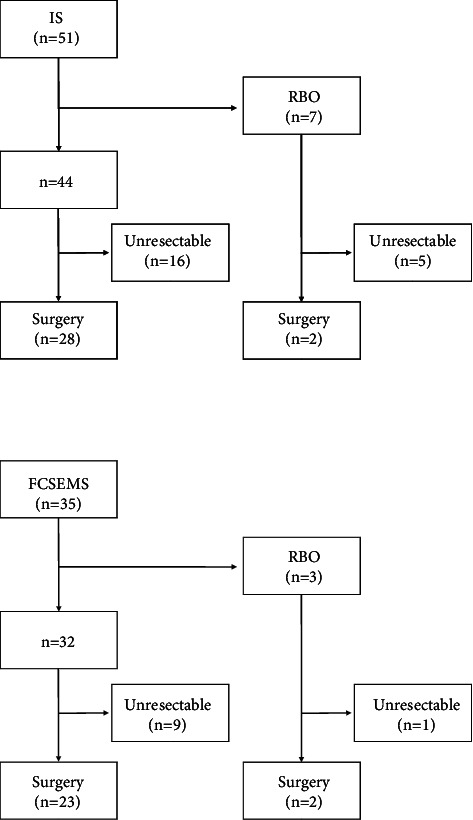
Clinical course for each type of stent. IS, inside stent; FCSEMS, fully covered self-expandable metallic stent; RBO, recurrent biliary obstruction.

Of the 51 patients in the IS group, 21 were diagnosed as unresectable and were treated with chemotherapy, radiation therapy, or palliative therapy. The reasons for unresectability were as follows: 16 patients due to disease progression during the course of treatment, 4 patients due to other diseases, and 1 patient due to poor liver function. Of the other 30 patients, 26 underwent hepatectomy of one or more lobes, 3 underwent hepatopancreatoduodenectomy, and 1 underwent pancreatoduodenectomy. Of the 35 patients in the FCSEMS group, 10 were diagnosed as unresectable due to disease progression; of the other 25, 14 underwent hepatectomy of one or more lobes, 5 underwent hepatopancreatoduodenectomy, 5 underwent pancreatoduodenectomy, and 1 underwent extrahepatic bile duct resection (Figure 2).

### 3.2. RBO and Time to RBO

A total of 7/51 patients in the IS group and 3/35 patients in the FCSEMS group developed RBO, with no significant difference between the groups (*P*=0.464). Among 44 patients in the IS group who did not develop RBO, 28 underwent surgery; the other 16 patients were diagnosed as unresectable. Among 32 patients in the FCSEMS group who did not develop RBO, 23 underwent surgery; the other 9 patients were diagnosed as unresectable (Figure 2). The median time (IQR) from FSPBD to operation was 41 (34–55) days in the IS group and 43 (33–58) days in the FCSEMS group (*P*=0.836). In all 7 patients who developed RBO in the IS group, the IS was removed and replaced with endoscopic nasobiliary drainage. For all 3 patients who developed RBO in the FCSEMS group, segmental cholangitis of the undrained area was the reason for RBO. The FCSEMS was removed, and two or three endoscopic nasobiliary drainages were inserted in both the future remnant and the excision liver lobe(s). Stent removal was performed easily and without complications in all patients.

**Figure 3:**
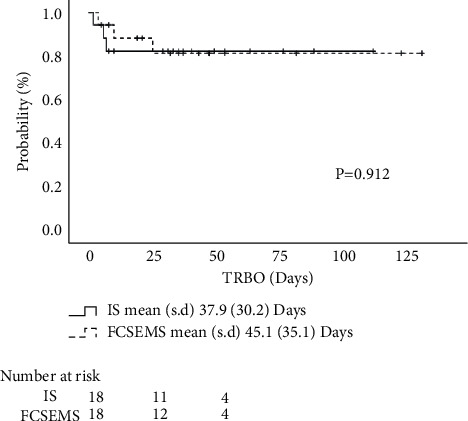
Time to RBO for each stent after propensity score matching. The mean (SD) time to RBO was 37.9 (30.2) days in the IS group and 45.1 (35.1) days in the FCSEMS group, with no significant difference (*P*=0.912, log-rank test). RBO, recurrent biliary obstruction; IS, inside stent; FCSEMS, fully covered self-expandable metallic stent.

Propensity score matching based on a propensity score was used to obtain a uniform Bismuth-Corlette classification I/II and III/IV in IS and FCSEMS groups. This resulted in the extraction of 18 patients from each group (Table 1). After propensity score matching, 3 of 18 patients resulted in RBO for both groups. Although the median time to RBO could not be reached, the mean (SD) time to RBO was 37.9 (30.2) days in the IS group and 45.1 (35.1) days in the FCSEMS group, with no significant difference (*P*=0.912, log-rank test) (Figure 3).

### 3.3. Factors Related to RBO

In multivariate analysis, obstruction of the bile duct branch was a significant risk factor only in the FCSEMS group (HR 29.8, 95% CI 2.5–350.1, *P*=0.008) (Table 2). In contrast, there was no significant factor in the IS group. In the absence of obstruction of the bile duct branch by stenting (17 patients in the IS group and 32 in the FCSEMS group), RBO occurred in only 1 patient in each group.

### 3.4. Adverse Events after ERC

Cholangitis without RBO occurred in 10/51 patients in the IS group and in 5/35 patients in the FCSEMS group (*P*=0.523). Cholecystitis was not diagnosed in the IS group but occurred in 1/35 patients in the FCSEMS group (*P*=0.225). Post-ERC pancreatitis occurred in 4/51 patients in the IS group and in 1/35 patients in the FCSEMS group (*P*=0.332); all cases of pancreatitis were mild and were cured with conservative treatment. There was no significant difference between the two groups in the incidence of these adverse events.

### 3.5. Postoperative Complications

A total of 28 patients in the IS group and 23 patients in the FCSEMS group who underwent surgery without RBO were evaluated. Hepatectomy, including hepatopancreatoduodenectomy, was more common in the IS group (27/28 patients) than in the FCSEMS group (17/23 patients) (*P*=0.020), whereas pancreatectomy, including hepatopancreatoduodenectomy, was more common in the FCSEMS group (11/23 patients) than in the IS group (4/28 patients) (*P*=0.009) (Table 3a). There was no significant difference in bleeding or operation time between the two groups. Regarding postoperative complications, there was no significant difference in the incidence of bile leakage or liver failure. However, pancreatic fistula was significantly more common in the FCSEMS group (13/23 patients) than in the IS group (3/28 patients) (*P*=0.001) (Table 3b).

There was no significant difference in the incidence of bile leakage between the two groups when patients were classified according to whether they had undergone hepatectomy (Table 4). Similarly, pancreatic fistula was classified according to whether the patient had undergone pancreatectomy (Table 5). In patients who underwent pancreatectomy, there was no significant difference between the IS and FCSEMS groups in the incidence of pancreatic fistula, but in patients who did not undergo pancreatectomy, the incidence of pancreatic fistula was significantly higher in the FCSEMS group (5/12 patients) than in the IS group (0/24 patients) (*P*=0.001).

## 4. Discussion

In the diagnosis of PHCC, contrast-enhanced multidetector-row computed tomography should be conducted after hematological examination and abdominal ultrasonography, enabling accurate tumor staging [3]. Based on this assessment, PBD should be performed in jaundiced patients who are scheduled for major hepatectomy [11, 12, 28, 29] because mortality remains high in this setting, mainly due to liver failure [3]. Also, because it is estimated that the risk of developing de novo malignancies after liver transplantation is high [30], it is expected that PBD will be performed more frequently in such patients in the future.

In recent years, the efficacy of stenting above the sphincter of Oddi has been highlighted as a new PBD approach for PHCC. The primary benefit of this approach retains the sphincter of Oddi [14], which works as a guardian of duodenobiliary reflux potentially triggering biliary contamination of enterobacteria. Another advantage is that multiple (2 to 3) stents can be placed without endoscopic sphincterotomy because stenting above the papilla does not compress the pancreatic duct orifice. Endoscopic sphincterotomy is a significant risk factor for RBO in endoscopic nasobiliary drainage [23], whereas endoscopic sphincterotomy was not a risk factor for RBO in IS or FCSEMS as shown in this study. A plausible explanation is difficult to provide for these contrasting observations, but it could be a favorable finding for patients who have undergone endoscopic sphincterotomy prior to IS or FCSEMS above the sphincter. The potential disadvantage of the nonendoscopic sphincterotomy policy is that stent removal may be difficult if stents are totally stored within the biliary system. However, in this study, such stents were successfully removed even in patients without endoscopic sphincterotomy.

In the current study, IS and FCSEMS were placed in patients at distances of more than 20 mm between the papilla and the lower end of the stricture. A distance of at least 20 mm between the papilla and the stenosis appears to be sufficient to enable stenting of the bile duct while retaining papillary function. Unlike distal malignant biliary obstruction, in PHCC, the drainage methods should be determined based on both the confluence of the bilateral bile ducts and the extension of the tumor. Bismuth I/II was more common in the FCSEMS group because FCSEMS is considered to be indicated when the confluence of the first bile duct of the future remaining liver lobe is more than 5 mm from the upper end of the stenosis.

In this study, obstruction of the bile duct branch in the future excisional liver lobe(s) by stenting was a risk factor for RBO only when FCSEMS was used. Furthermore, there was no significant difference between FCSEMS and IS in RBO rate, even in patients in whom the bile duct branch had not been obstructed by stenting. Therefore, when there is a risk of obstruction of the bile duct branch by stenting, it is not necessary to place FCSEMS, and placing the IS is sufficient for PBD.

It is thought that the use of uncovered self-expandable metallic stents for PBD of PHCC induces periductal fibrosis, enhancing the difficulty of surgical resection, and that uncovered self-expandable metallic stents should therefore not be recommended if there is any possibility of resection [31]. However, caution should be paid that those opinions exclusively used large-bore uncovered self-expandable metallic stents, not small-bore FCSEMSs that were studied in the present study. The attending surgeons in the present study did not complain about FCSEMS because periductal fibrosis was within an acceptable limit, although an objective scoring system was not utilized. In addition, operative outcomes and most postoperative complications were similar between the IS and FCSEMS groups, except for pancreatic fistula. Pancreatic fistula is a cautionary complication because it can lead to more severe consequences such as pseudoaneurysms, bleeding, tissue necrosis, and abscess formation [32]. Watanabe et al. reported a general incidence of pancreatic fistula (grade B/C on International Study Group of Pancreatic Surgery) rate of 10.7% after PHCC surgery, which was closely related to a specific surgical maneuver intrapancreatic duct resection [31, 33]. In the present series without pancreatectomy, the incidence of pancreatic fistula in the FCSEMS group was much higher than that in the IS group. This great difference may be explained by FCSEMS overexpansion with subsequent periductal fibrosis around the pancreatic entry, which complicates the step of distal division of the bile duct and peripancreatic lymph node dissection; further studies are needed to address this surgical matter.

The limitations of this study include its retrospective and single-center nature with a small sample size. In particular, as stent selection was not clearly predefined, heterogeneous patient background, such as in the Bismuth-Corlette classification, suggests a careful interpretation of the present results. Regardless, this limitation is unlikely to have affected the result that the RBO rate in the IS group, which encompasses patients whose conditions are considered to be worse, was equal to that in the FCSEMS group or the result indicating that there were fewer cases of postoperative pancreatic fistula in the IS group. Because this was not a multicenter study and the number of cases is not huge, it is difficult to draw any conclusions, but more cases and studies are needed in the future.

In conclusion of this study, as PBD for PHCC, both IS and FCSEMS achieved low RBO rates, and there was no significant difference in RBO rate between the IS and FCSEMS groups. In contrast, the incidence of postoperative pancreatic fistula was higher with FCSEMS. Thus, IS, which can be inserted easily, is considered an optimal approach as PBD for resectable PHCC.

## Figures and Tables

**Table 1:** Clinical characteristics according to the type of stent.

Variables	All patients			Propensity matched patients		
	IS (*n* = 51)	FCSEMS (*n* = 35)	*P* value	IS (*n* = 18)	FCSEMS (*n* = 18)	*P* value
Age, *y* (range)	70 (25–84)	71 (46–83)	0.273	70 (52–80)	71 (46–83)	0.960
Sex, *n* (%)			0.316			0.717
Male	36 (70.6)	28 (80.0)		13 (72.2)	12 (80.0)	
Female	15 (29.4)	7 (20.0)		5 (27.8)	6 (20.0)	
Bismuth-Corlette classification, *n* (%)			<0.001			1.000
I/II	9 (17.6)	26 (74.3)		9 (50.0)	9 (50.0)	
III/IV	42 (82.4)	9 (25.7)		9 (50.0)	9 (50.0)	
Primary carcinoma, *n* (%)			0.439			0.674
Bile duct	44 (86.3)	28 (80.0)		14 (77.8)	15 (80.0)	
Gallbladder	7 (13.7)	7 (20.0)		4 (22.2)	3 (20.0)	
Previous drainage (total), *n* (%)			0.109			0.206
With	40 (78.4)	32 (91.4)		13 (72.2)	16 (88.9)	
Without	11 (21.6)	3 (8.6)		5 (27.8)	2 (11.1)	
Total bilirubin before ERCP, *n* (%)			0.338			0.289
Over 3.0 mg/dl	4 (7.8)	5 (14.2)		1 (5.6)	3 (16.7)	
Under 3.0 mg/dl	47 (92.2)	30 (85.8)		17 (94.4)	15 (83.3)	
Previous endoscopic sphincterotomy, *n* (%)			0.348			0.738
With	21 (41.2)	18 (51.4)		8 (44.4)	9 (50.0)	
Without	30 (58.8)	17 (48.6)		10 (55.6)	9 (50.0)	

^*∗*^There are duplicates. IS, inside stent; FCSEMS, fully covered self-expandable metallic stent.

**Table 2:** Univariable and multivariable analyses of reinterventions.

(2a) IS				
Variable	Reintervention		*P* value	
	With (*n* = 7)	Without (*n* = 44)	Univariate	Multivariate

Bismuth-Corlette classification, *n* (%)			0.187	0.309
I/II	0 (0.0)	9 (20.4)		
III/IV	7 (100.0)	35 (79.6)		
Previous endoscopic sphincterotomy, *n* (%)			0.355	
With	4 (57.1)	17 (38.6)		
Without	3 (42.9)	27 (61.4)		
Stenosis length over 20 mm, *n* (%)			0.402	
With	5 (71.4)	24 (54.5)		
Without	2 (28.6)	20 (45.5)		
Obstruction of the bile duct branch, *n* (%)			0.250	
With	6 (85.7)	28 (63.6)		
Without	1 (14.3)	16 (36.4)		
Distance of over 10 mm from stenosis to the first branch, *n* (%)			0.401	
With	1 (14.3)	13 (29.5)		
Without	6 (85.7)	31 (70.5)		

(2b) FCSEMS				
Variable	Reintervention		*P* value	
	With (*n* = 3)	Without (*n* = 32)	Univariate	Multivariate

Bismuth-Corlette classification, *n* (%)			0.002	0.068
I/II	0 (0.0)	26 (81.2)		
III/IV	3 (100.0)	6 (18.8)		
Previous endoscopic sphincterotomy, *n* (%)			0.581	
With	2 (66.7)	16 (50.0)		
Without	1 (33.3)	16 (50.0)		
Stenosis length over 20 mm, *n* (%)			0.805	
With	2 (66.7)	19 (59.4)		
Without	1 (33.3)	13 (40.6)		
Obstruction of the bile duct branch, *n* (%)			<0.001	0.008; HR 29.8, 95%CI 2.5–350.1
With	2 (66.7)	1 (3.1)		
Without	1 (33.3)	31 (96.9)		
Distance of over 10 mm from stenosis to the first branch, *n* (%)			0.027	0.127
With	0 (0.0)	21 (65.6)		
Without	3 (100.0)	11 (34.4)		

IS, inside stent; FCSEMS, fully covered self-expandable metallic stent.

**Table 3:** Patients without reintervention.

Variables	IS (*n* = 28)	FCSEMS (*n* = 23)	*P* value
*(a) Operative procedure*			
Hepatectomy			0.020
With	27	17	
L2	3	1	
L2 + PD	1	1	
L3	9	0	
L3 + PD	0	2	
R2	9	8	
R2 + PD	1	3	
R3	3	2	
R3 + PD	1	0	
Without	1	6	
Choledochectomy	0	1	
PD	1	5	
Pancreatectomy			0.009
With	4	11	
Without	24	12	

*(b) Postoperative course and complications*			
Operation time (min)	510 (479–610)	571 (513–615)	0.289
Bleeding (ml)	834 (638–601)	919 (677–1230)	0.470
Bile leakage, *n* (%)	14 (50.0)	8 (34.8)	0.275
Grade B	14	8	
Grade C	0	0	
Pancreatic fistula, *n* (%)	3 (10.7)	13 (56.5)	<0.001
Grade B	3	13	
Grade C	0	0	
Liver failure, *n* (%)	2 (7.1)	0 (0.0)	0.191
Grade B	1	0	
Grade C	1	0	

IS, inside stent; FCSEMS, fully covered self-expandable metallic stent; L2, left hepatectomy; L3, left hepatic trisectionectomy; R2, right hepatectomy; R3, right hepatic trisectionectomy; PD, pancreatoduodenectomy.

**Table 4:** Postoperative complications of bile leakage classified with and without hepatectomy and pancreatectomy.

Bile leakage	With (*n* = 22)	Without (*n* = 29)	*P* value
With hepatectomy	22	22	0.757
IS	14	13	
FCSEMS	8	9	

Without hepatectomy	0	7	Not available
IS	0	1	
FCSEMS	0	6	

IS, inside stent; FCSEMS, fully covered self-expandable metallic stent.

**Table 5:** Postoperative complications of pancreatic fistula classified with and without hepatectomy and pancreatectomy.

Pancreatic fistula	With (*n* = 16)	Without (*n* = 35)	*P* value
With pancreatectomy	11	4	0.930
IS	3	1	
FCSEMS	8	3	

Without pancreatectomy	5	31	0.001
IS	0	24	
FCSEMS	5	7	

IS, inside stent; FCSEMS, fully covered self-expandable metallic stent.

## Data Availability

The data used to support the findings of this study are available from the corresponding author upon request.

## Abbreviations

PBD: Preoperative biliary drainage
IS: Inside stent
FCSEMS: Fully covered self-expandable metallic stent
PHCC: Perihilar cholangiocarcinoma
RBO: Recurrent biliary obstruction
FSPBD: Final scheduled preoperative biliary drainage
ERC: Endoscopic retrograde cholangiography.
